# Nucleoside 5′-*O*-monophosphorothioates as modulators of the P2Y14 receptor and mast cell degranulation

**DOI:** 10.18632/oncotarget.12541

**Published:** 2016-10-09

**Authors:** Edyta Gendaszewska-Darmach, Edyta Węgłowska, Aurelia Walczak-Drzewiecka, Kaja Karaś

**Affiliations:** ^1^ Institute of Technical Biochemistry, Faculty of Biotechnology and Food Sciences, Lodz University of Technology, Stefanowskiego, Lodz, Poland; ^2^ Laboratory of Cellular Immunology, Institute of Medical Biology, Polish Academy of Sciences, Lodowa, Lodz, Poland

**Keywords:** P2Y receptors, nucleoside 5'-O-monophosphorothioates, inflammation, degranulation, RBL-2H3 cells, Immunology and Microbiology Section, Immune response, Immunity

## Abstract

Mast cells (MCs) are long-lived resident cells known for their substantial role in antigen-induced anaphylaxis and other immunoglobulin E-mediated allergic reactions as well as tumor promotion. MCs' activation results in the release of pro-inflammatory factors such as histamine, tryptase, tumor necrosis factor or carboxypeptidase A stored in secretory granules. IgE-dependent hypersensitivity has been thought to be the major pathway mediating degranulation of mast cells, but the P2Y14 nucleotide receptor activated by UDP-glucose (UDPG) may also enhance this process. In this study we identified thymidine 5'-*O*-monophosphorothioate (TMPS) as a molecule inhibiting UDPG-induced degranulation in a rat mast cell line (RBL-2H3). Additionally, TMPS diminished UDPG-evoked intracellular calcium mobilization in a stable HEK293T cell line overexpressing the P2Y14 receptor. Therefore, we demonstrate that the use of thymidine 5'-*O*-monophosphorothioate might be a novel anti-inflammatory approach based on preventingmast cell activation.

## INTRODUCTION

Mast cells (MCs) are a population of leukocytes derived from haematopoietic progenitor cells that are associated with hypersensitivity reactions. Once activated, mast cells release a lot of pro-inflammatory cytokines, including histamine, tumor necrosis factor, and serotonin stored in secretory granules [1]. These are critical agents in the pathogenesis of asthma, allergic rhinitis, mastocytosis, psoriasis, and the progression of numerous cancers [2]. An association between chronic inflammation and increased susceptibility to neoplastic transformation has been documented for many years [3]. Circulating mast cells precursors migrate into the developing tumor [4] and increased number of MCs was found in human lymphoid neoplasms [5], thyroid [6], prostate [7] or pancreatic cancers [8]. It has been also shown that histamine and chemokines (CXCL1/GRO-α, CXCL10/IP-10, and CXCL8/IL-8) released by activated human MCs modulate proliferation, survival, and invasion of thyroid cancer cells through the involvement of specific receptors [9]. Thus, specific inhibitors focused on preventing mast cell activation might prospectively offer novel anti-inflammatory therapeutic approaches.

Degranulation of mast cells can be evoked by immunoglobulin E (IgE) and non-immunologic agents [2]. Activation of high-affinity Ig E receptor (FcεRI) plays an extremely important role in the release of pro-inflammatory mediators [2, 10]. However, in a physiological setting, other receptors, such as P2Y family activated by nucleotides, might also markedly influence the release of mediators by mast cells [11, 12]. P2Y nucleotide receptors (P2YR), belonging to G protein-coupled receptors (GPCRs) include eight subtypes, namely P2Y1, P2Y2, P2Y4, P2Y6, P2Y11, P2Y12, P2Y13 and P2Y14 [13]. P2Y1, P2Y12 and P2Y13 receptors respond to ADP (ATP may be a partial agonist), but not to uracil nucleotides. P2Y2 is activated by both ATP and UTP, whereas P2Y6 preferentially by UDP. UTP is the P2Y4 agonist while ATP activates the rat homologue of this receptor and antagonizes the human homologue [14, 15]. P2Y11 receptor is activated primarily by ATP, while P2Y14 responds to the nucleotide sugar conjugate UDP-glucose (UDPG) and UDP [16, 17]. It has been shown that UDP-glucose enhances antigen-induced release of *N*-acetyl-β-D-hexosaminidase (a granule enzyme that parallels histamine release) in rat RBL-2H3 and human LAD2 mast cells *via* the P2Y14R [11, 12]. P2Y14 receptor is involved not only in inflammatory, but also hypoxic and endocrine signalling and thus serves as an attractive pharmacological target [18, 19].

The P2Y14R has been considered a nucleotide sugar-activated purinergic receptor with a following order of potency observed: UDP-glucose > UDP-galactose > UDP-glucuronic acid > UDP-N-acetylglucosamine [20, 21, 22]. Chambers et al. reported that ADP-glucose, UMP, UDP, and UTP as well as uridine were unable to activate P2Y14 [21, 23]. The discovery by Carter et al. that UDP also acts as a potent agonist revealed that a sugar moiety is not required for activation of the P2Y14 receptor. In those studies slight activities were observed with ADP, CDP, and GDP at concentrations greater than 100-fold higher than the concentrations of UDP producing a maximal response [22, 24]. Hamel et al. [23] showed with conventional and nonconventional methods that even UMP selectively activated HEK cells coexpressing P2Y14 and Gαqi5. The presence of 2-thiouracil modification within UDPG and UDP amplified potency at the human P2Y14 receptor with an EC_50_ value of 11 nm and 1.92 nM, respectively, as compared to UDPG (EC_50_ 400 nM) and UDP (EC_50_ 160 nM) [25, 26].

**Figure 1:**
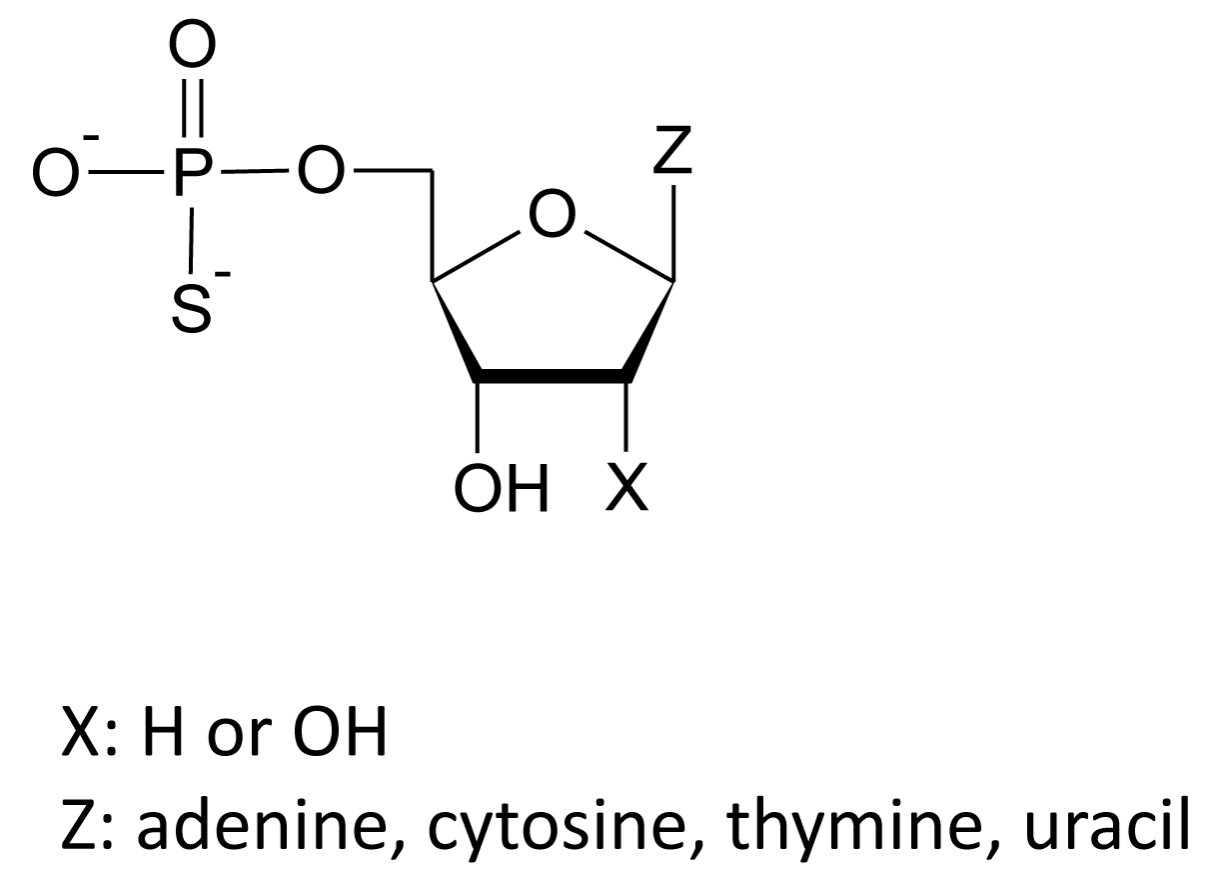
Structures of nucleoside 5′-*O*-monophosphorothioate analogues studied

Recently we have shown that nucleoside 5'-*O*-monophosphorothioate analogues, containing a sulfur atom in place of one of non-bridging oxygen atoms in a phosphate group (Figure 1), can act as ligands for P2Y6 receptor. We paid particular attention to the unique activity of thymidine 5'-*O*-monophosphorothioate (TMPS) which acts as a selective partial agonist of the P2Y6R [27]. Here we show that in HEK293T cells stably expressing P2Y14R uridine 5'-*O*-monophosphorothioate (UMPS), cytidine 5'-*O*-monophosphorothioate (CMPS), and adenosine 5'-*O*-monophosphorothioate (AMPS) act as P2Y14 agonists while TMPS serves as an antagonist. Using rat basophilic leukemia RBL-2H3 cells where the P2Y14 receptor is endogenously expressed [11], we demonstrate that TMPS does not induce degranulation in opposition to UDPG, UMPS, CMPS, AMPS, and a selective P2Y14 receptor agonist, namely MRS 2690 (diphosphoric acid 1-α-D-glucopyranosyl ester 2-[(4'-methylthio)uridin-5''-yl] ester). Moreover, UDPG- and MRS 2690-induced increase of *N*-acetyl-β-D-hexosaminidase release can be blocked by TMPS pre-treatment. It may suggest that thymidine 5'-O-monophosphorothioate possesses unique activity to antagonize the P2Y14 receptor and inhibit mast cell degranulation. An additional advantage for the therapeutic potential of nucleoside 5'-O-monophosphorothioates is that replacing an oxygen atom with a sulfur in a phosphate group confers a significant resistance toward enzymatic hydrolysis catalyzed by numerous enzymes, including the ectonucleotide pyrophosphatase/phosphodiesterase family, the ecto-nucleoside triphosphate diphosphohydrolase family, and ecto-5'-nucleotidases [27, 28]. A phosphorothioate modification of nucleotides substantially prolongs their half-life and duration of action.

## RESULTS

### Determination of RBL-2H3 cells viability

**Figure 2:**
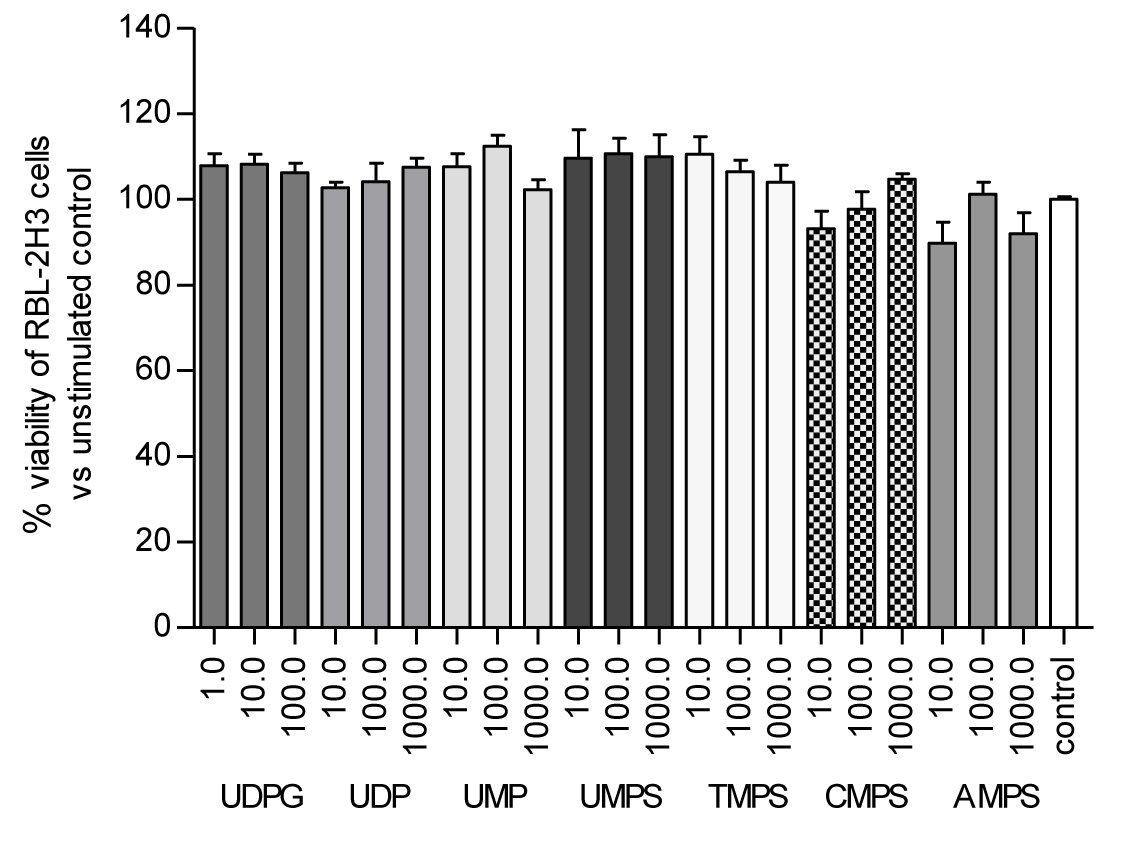
The effect of UDPG (1.0 μM; 10.0 μM; 100.0 μM), UMP and nucleoside 5′-*O*-monophosphorothioates (10.0 μM; 100.0 μM; 1000.0 μM) on the viability of RBL-2H3 cells Viability was assessed using PrestoBlue kit after 24 h of incubation. Data represent the means ± SD from at least three independent experiments; *p < 0.05 vs unnstimulated control cells (100%).

Firstly, we determined the effects of nucleoside 5'-*O*-monophosphates and nucleoside 5'-O-monophosphorothioates on RBL-2H3 cell viability using a rezasurin-based assay. We compared the effects of unmodified uridine-containing P2Y14 agonists, namely UDP-glucose, UDP and UMP. Since we have recently shown that nucleoside 5'-*O*-monophosphorothioate analogues could act as P2Y receptor ligands, we also tested pyrimidine nucleotides (UMPS, TMPS, CMPS) serving as prime subjects of this research and adenosine 5'-*O*-monophosphorothioate (AMPS) as a purine control. After 24 hours of incubation all the compounds tested did not significantly inhibit cellular viability (Figure 2). Thus, one can assume that UDP-glucose and nucleoside 5'-*O*-monophosphorothioate analogues do not possess cytotoxic properties exhibiting good safety profile even at the highest concentration used, namely 100 μM and 1000 μM.

### Inhibitory effects of thymidine 5'-*O*- monophosphorothioate on UDPG- and MRS 2690-induced β-HEX release from RBL-2H3 cells

Gao et al. previously showed that the P2Y14 receptor agonist (UDPG) alone was unable to induce *N*-acetyl-β-D-hexosaminidase (β-HEX) release but it concentration-dependently enhanced antigen-induced degranulation [11]. β-HEX release was also used in our studies as an indicator of the RBL-2H3 degranulation. Therefore we first determined the relationship between FcεR-mediated cell degranulation and the concentration of the antigen used (DNP-HSA). RBL-2H3 cells were primed with increasing concentrations of the antigen (1 - 1000 ng/ml) and the maximal β-HEX release (29.46 ± 9.92%) was observed for DNP-HSA used at the concentration of 100 ng/ml. Thus for further experiments submaximal concentration (10 ng/ml) of the antigen was chosen which proved to stimulate degranulation at the level of 6.28 ± 4.76% (Figure S1; Supplementary Materials).

**Figure 3:**
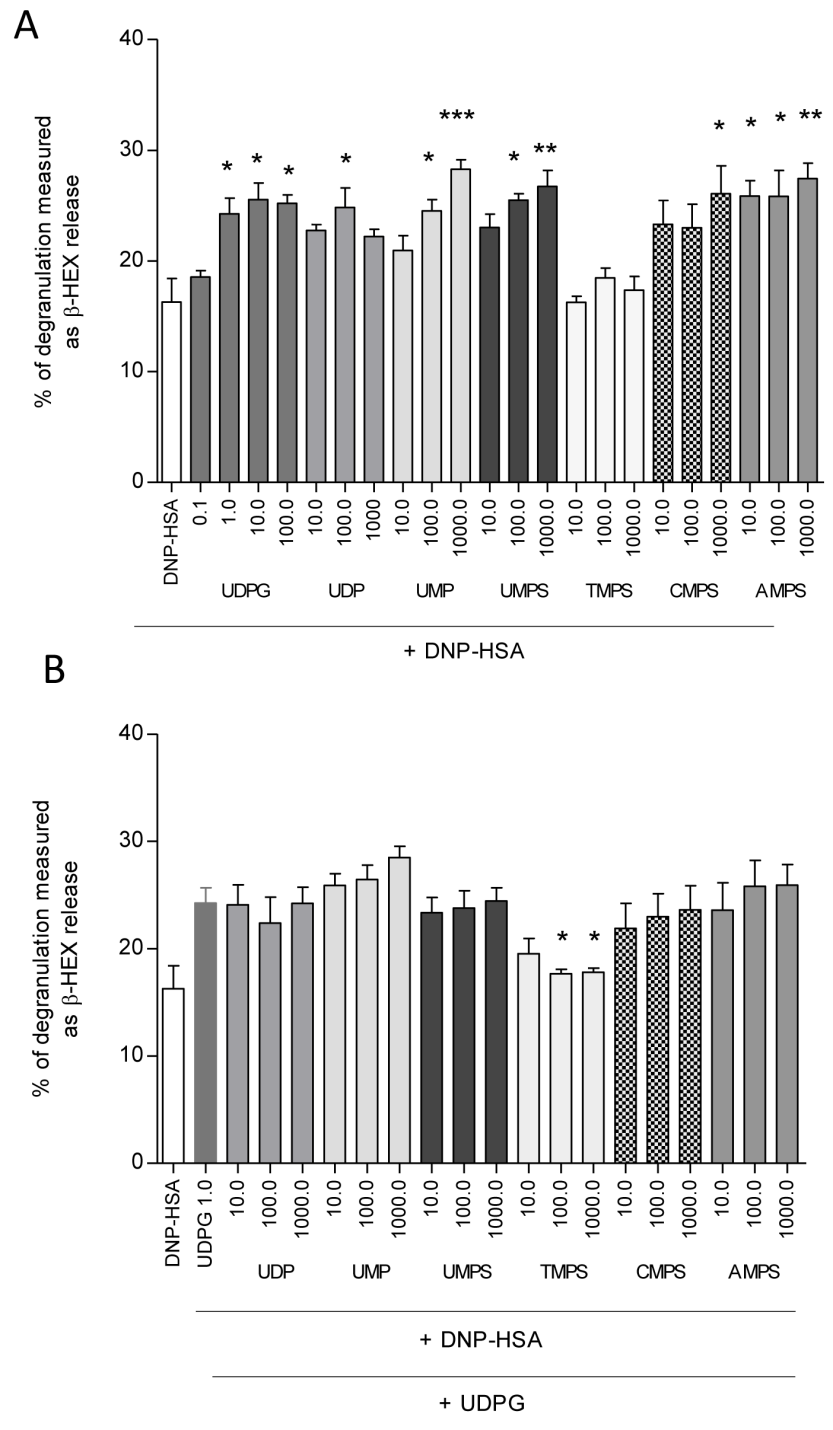
The influence of UDPG (0.1 μM; 1.0 μM; 10.0 μM; 100.0 μM), UMP and nucleoside 5′-*O*-monophosphorothioates (10.0 μM; 100.0 μM; 1000.0 μM) on β-HEX release from RBL-2H3 cells Mast cells were sensitized for 24 h with DNP-specific IgE antibody (0,5 μg/ml) and incubated for 20 min with antigen in the presence of tested nucleotides used at the concentration of **A.** For antagonist assay, cells were pre-incubated with the compounds (10.0 μM; 100.0 μM; 1000.0 μM) for 10 minutes before the application of UDPG (1.0 μM) serving as the control agonist **B.** The spontaneous release (9.5%) was subtracted from the final data. Data represent the means ± SD from at least three independent experiments; *p < 0.05, **p < 0.1. ***p < 0.001 when compared to DNP-HSA (A) or DNP-HSA with 1.0 μM UDPG (B).

Then we examined the influence of nucleotides on DNP-HSA-mediated β-HEX release in RBL-2H3 cells. As expected UDP-glucose significantly elevated antigen-induced degranulation. The maximal response was observed in the case of 10 μM UDPG inducing degranulation at the level of 25.55 ± 4.24% (Figure 3A). UDP, UMP, UMPS, CMPS, and AMPS also caused enhancement of β-HEX release. The only exception was TMPS in which case even at 1000 μM concentration no degranulation over antigen alone was observed (Figure 3A). It might have suggested that UMPS, CMPS, and AMPS but not TMPS could activate the P2Y14 receptor.

**Figure 4:**
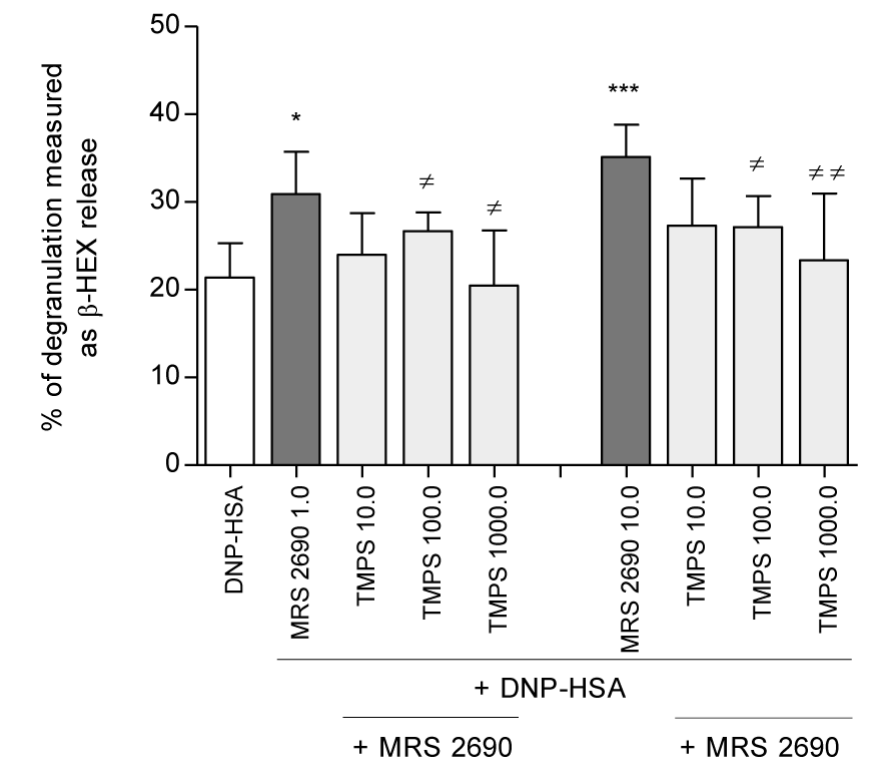
The influence of TMPS pre-treatment (10.0 μM; 100.0 μM; 1000.0 μM) on MRS 2690-induced (1.0 μM; 10.0 μM) β-HEX release from RBL-2H3 cells Mast cells were sensitized for 24 h with DNP-specific IgE antibody (0,5 μg/ml)and pre-incubated with TMPS for 10 minutes before the application of MRS 2690 serving as the control agonist. The spontaneous release (9.5%) was subtracted from the final data. Data represent the means ± SD from at least three independent experiments; * *p* < 0.05, *** *p* < 0.001 when compared to DNP-HSA or ≠*p* < 0.05, ≠≠ *p* < 0.01 when compared to MRS 2690.

During the next step cells were pre-incubated with the nucleotides tested for 10 minutes before the application of UDPG serving as the P2Y14 agonist. UDPG-induced increase of RBL-2H3 cell degranulation was blocked only in the case of pre-treatment with TMPS. Other nucleotides applied did not influence β-HEX release (Figure 3B). These results suggest that thymidine 5'-*O*-monophosphorothioate may antagonize the P2Y14 receptor. To examine whether inhibition RBL-2H3 cell degranulation by TMPS was specifically mediated *via* the P2Y14 we used a selective P2Y14 receptor agonist, namely MRS 2690 (diphosphoric acid 1-α-D-glucopyranosyl ester 2-[(4'-methylthio)uridin-5''-yl] ester) possessing 7-fold higher potency than UDPG [25]. Figure 4 shows that MRS 2690 itself increased antigen-induced β-HEX release in a concentration-dependent manner and 10-minute pre-treatment with TMPS significantly diminished MRS 2690 caused enhancement. The results have proved that thymidine 5'-*O*-monophosphorothioate plays an important role in inhibiting mast cell degranulation *via* antagonizing of the P2Y14 receptor

### Intracellular calcium mobilization assay in RBL-2H3 cells

**Figure 5:**
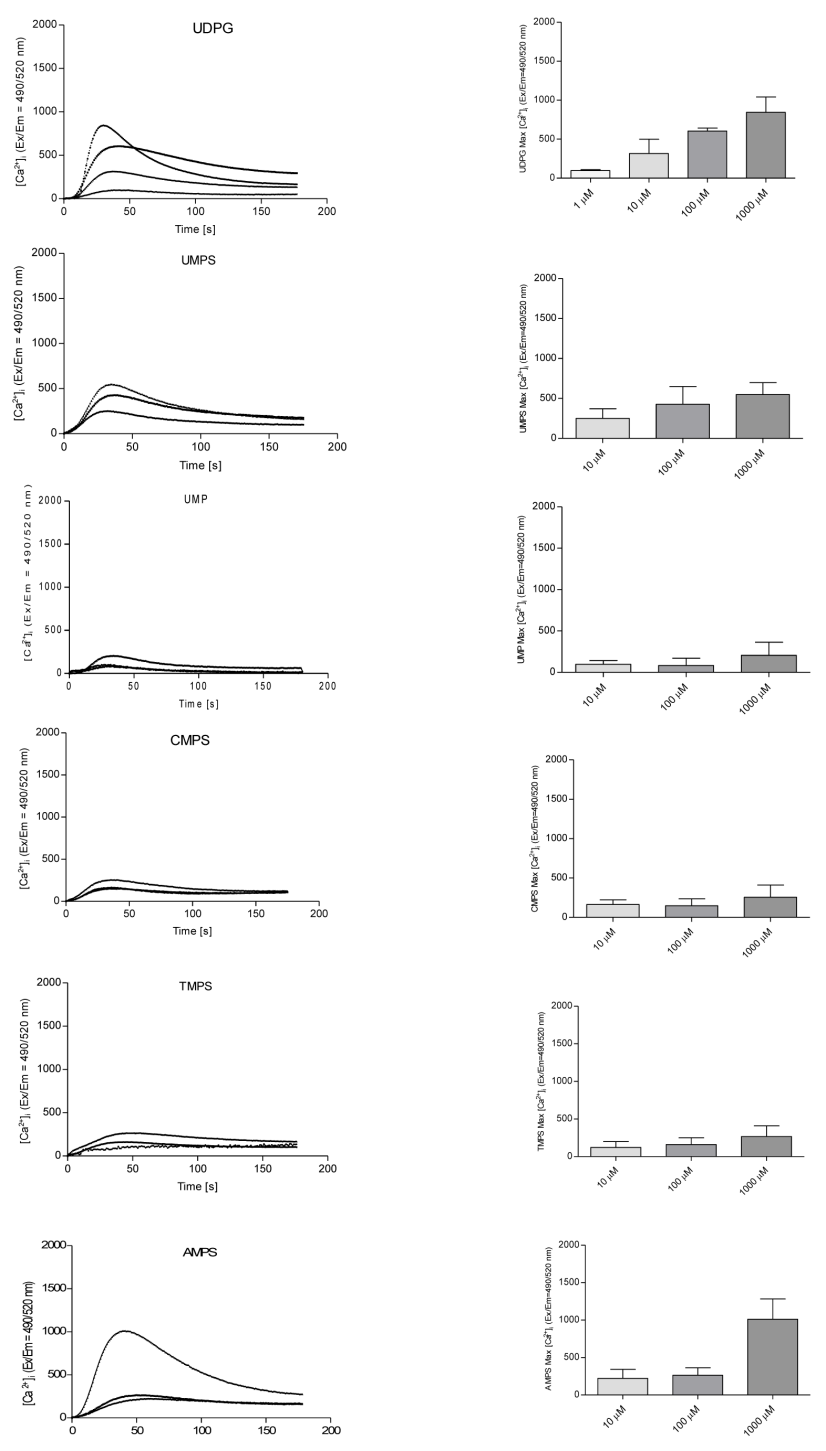
Stimulation of intracellular calcium mobilization in RBL-2H3 cells by UDPG (1.0 μM; 10.0 μM; 100.0 μM; 1000.0 μM), UMP and nucleoside 5'-O-monophosphorothioates (10.0 μM; 100.0 μM; 1000.0 μM) Left panel represents time course of mean Ca^2+^ responses (Fluo-8 ratio) and right panel demonstrates the maximal fluorescence intensity obtained from traces shown in the left panel. Obtained results represent the means from at least three independent experiments.

Gao et al. [11] showed that UDP-glucose not only enhanced degranulation process but also elevated intracellular calcium ions level with EC_50_value of 5980 ± 1140 nM in RBL-2H3 mast cells. We applied a Fluo-8 probe to determine [Ca^2+^]_i_ changes upon nucleoside 5'-*O*-monophosphorothioates treatment. The relative fluorescent units (RFU) were monitored for 180 seconds. In our studies stimulation with UDPG as well as AMPS, UMPS, CMPS and TMPS increased cytosolic Ca^2+^ concentration. The peak heights were parallel with the final nucleotide concentration. At 1 mM concentration adenosine 5'-*O*-monophosphorothioate evoked the highest elevation of [Ca^2+^]_i_ followed by UDPG, UMPS, TMPS, and CMPS. UMP was also shown to induce intracellular calcium flux, but to a lower extend (Figure 5).

**Figure 6:**
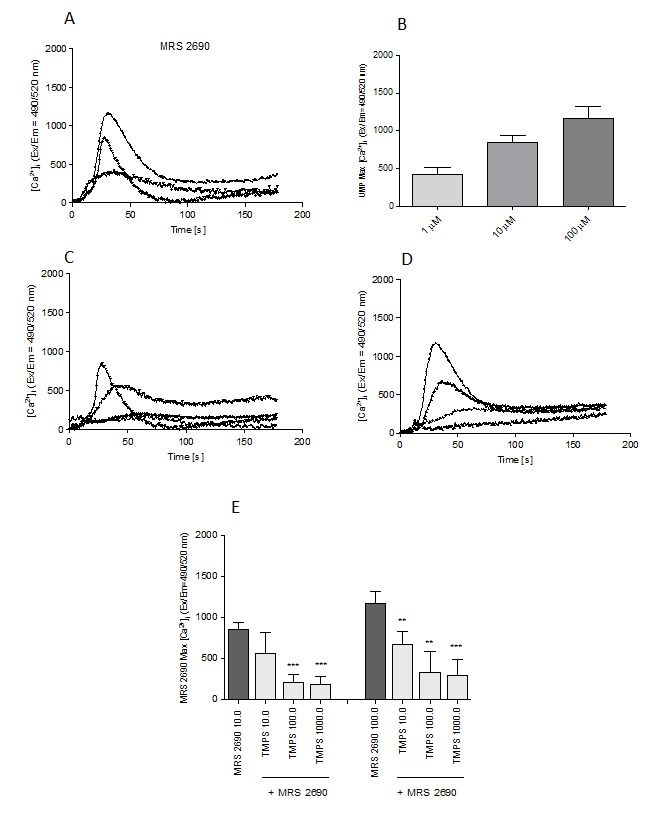
Stimulation of intracellular calcium mobilization in RBL-2H3 cells by MRS 2690 (1.0 μM; 10.0 μM; 100.0 μM) and the effect of TMPS pre-treatment (10.0 μM; 100.0 μM; 1000.0 μM) **A.** Time course of mean Ca^2+^ responses (Fluo-8 ratio) after MRS 2690 application; **B.** The maximal fluorescence intensity obtained from traces shown in A; **C.** The influence of TMPS pre-treatment on MRS 2690-induced (10.0 μM) calcium mobilization; **D.** The influence of TMPS pre-treatment on MRS 2690-induced (100.0 μM) calcium mobilization; **E.** the maximal fluorescence intensity obtained from traces shown in C and D. Data represent the means ± SD from at least three independent experiments; * *p* < 0.05, ** *p* < 0.01 *** *p* < 0.001 when compared to MRS 2690.

Among nucleoside 5'-O- monophosphorothioates tested in this study only TMPS behaved as an inhibitor of degranulation process. Therefore we employed once again the selective P2Y14 agonist and pre-incubated RBL-2H3 cells with thymidine 5'-O- monophosphorothioate before applying MRS 2690 and measuring intracellular calcium concentration. Figure 6 shows that TMPS treatment significantly blunted the increase of intracellular Ca^2+^ concentration evoked by MRS 2690, consistently with an antagonist function of TMPS for the P2Y14R.

### Calcium mobilization assay in HEK293T cells stably expressing individual P2Y14 receptor

To prove ultimately that nucleoside 5'-*O*- monophosphorothioates are ligands for the P2Y14 receptor a stable HEK293T cell line co-expressing the P2Y14R and the chimeric G-protein Gαqi5 was employed. Cells were probed with AMPS, UMPS, CMPS and TMPS to monitor stimulation of intracellular calcium mobilization. Surprisingly, among the nucleotide analogues studied at the concentration of 1 mM the most potent appeared to be a purine nucleotide, namely adenosine 5'-*O*-monophosphorothioate with a maximal response corresponding to 102.5 ± 7.1% of the maximal response induced by 1.0 μM UDPG. The rank order of potency was as follows: AMPS > UMPS > CMPS. The treatment with 1 mM TMPS failed to elicit a substantial calcium response from the stable HEK293T cell line expressing P2Y14R. At lower concentrations the most potent nucleotide was uridine 5'-*O*-monophosphorothioate, e.g. when 1.0 μM concentration was applied the observed effect corresponded to 84.5 ± 5.3% of the maximal response induced by 10.0 μM UDPG (Table 1).

**Table 1:** Nucleoside 5'-*O*-monophosphorothioates-dependent intracellular calcium response in HEK293T cells stably expressing human P2Y14 receptor (% activation as compared to the maximal response evoked by control agonist UDPG used at a concentration of 1.0 μM)

NUCLEOTIDE	CONCENTRATION			
	1000 μM	100 μM	10 μM	1 μM
TMPS	7.60 ± 5.38	NE	NE	NE
AMPS	102.5 ± 7.14*	84.4 ± 17.63*	33.6 ± 15.81*	6.8 ± 3.35
UMPS	91.8 ± 2.08*	89.1 ± 6.47*	80.7 ± 12.97*	84.5 ± 5.34*
CMPS	72.6 ± 5.81*	77.5 ± 4.40*	NE	NE

Calcium mobilization assay in HEK293T cells expressing human P2Y14 receptor was performed by Multispan Inc. (details in Materials and Methods). NE: no effect. Data represent the average ± SD of at least triplicate determinations.

* *p*<0.05 test

In antagonist assay, AMPS, UMPS and CMPS displayed dose-dependent antagonist activity that could be due to desensitization of the receptor since the earlier studies without pre-incubation did not show antagonist activity. At 10 μM concentration applied CMPS did not show any antagonistic activity in opposite to AMPS and UMPS lowering UDPG response to the level of 55.8 ± 21.2% and 43.8 ± 8.7%, respectively. The most interesting effect was observed in the case of thymidine 5'-*O*-monophosphorothioate that inhibited UDPG-induced effect despite the lack of P2Y14R agonist activity (Table 2). These results confirm once again that TMPS might act as an antagonist for the P2Y14 receptor.

**Table 2:** UDPG-induced intracellular calcium response in HEK293T cells stably expressing human P2Y14 receptor after 10-minute pre-treatment with nucleoside 5'-*O*-monophosphorothioates (% activation as compared to the 100% effect evoked by a control agonist used at a concentration of EC_80_ concentration corresponding to 0.22 μM)

NUCLEOTIDE	CONCENTRATION			
	1000 μM	100 μM	10 μM	1 μM
TMPS	17.30 ± 52.94*	72.30 ± 15.73*	75.41 ± 16.61*	98.83 ± 8.67
AMPS	5.71 ± 1.84*	1.51 ± 0.51*	55.80 ± 21.19*	93.72 ± 1.02
UMPS	35.60 ± 7.81*	37.0 ± 8.82*	43.80 ± 8.70*	47.31 ± 11.46*
CMPS	15.0 ± 1.33*	12.1 ± 1.60*	97.70 ± 9.84	96.63 ± 8.67

Calcium mobilization assay in HEK293T cells expressing human P2Y14 receptor was performed by Multispan Inc. (details in Materials and Methods). Data represent the average ± SD of at least triplicate determinations.

* *p*<0.05 test

Discussion

Several studies indicate a pro-inflammatory role for the P2Y14 receptor found on its high expression level in immune cells, and on the increased release of UDP-glucose, by damaged cells [19, 29, 30]. UDPG enhances the mobility of neutrophils [31, 32] and expression of P2Y14R in rat brain was shown to be up-regulated by immunologic challenge with lipopolysaccharide [33]. Besides, UDP-glucose release from hepatocellular injury may trigger innate immune responses and promote the development of insulin resistance [34]. What is more, UDP-glucose was shown to occur at high levels in cancer cells [35]. Thus, UDP-glucose is regarded as a pro-inflammatory factor and novel therapeutic strategies that inhibit P2Y14R may prove to be protective to the development of inflammatory processes.

The P2Y14R has been deliberated a nucleotide sugar-activated receptor for which UDP-glucose is the most active ligand [30, 36]. In contrast to other studies Carter et al. demonstrated that uridine diphosphate acted also as agonist of human P2Y14R [22]. Intracellular calcium mobilization and nonconventional cellular impedance functional assays showed that UMP, UDP, and UTP uncoupled to glucose also activated P2Y14-expressing cells [23].

Non-nucleotide dihydropyridopyrimidine [37] and naphthoic acid-containing [38] molecules were developed as potential P2Y14R inhibitors. Optimization of the latter compound revealed that 4-((piperidin-4-yl)-phenyl)-7-(4-(trifluoromethyl)-phenyl)-2-naphthoic acid (PPTN) acts as a high-affinity competitive antagonist of the P2Y14R [39]. Unfortunately, further studies demonstrated that PPTN had a high affinity towards serum proteins [40, 41]. Despite the fact that the 2-naphthoic acid class displays poor drug-like characteristics due to high molecular weight and high lipophilicity, such compounds can be used to develop high-affinity P2Y14R fluorescent probes for receptor detection and characterization [42].

Nucleoside 5'-*O*-monophosphates were not thought to act as agonists of the nucleotide receptors. However, Hamel et al. demonstrated that UMP selectively activated the P2Y14 receptor, although nucleotide diphosphates were more potent than monophosphates. The rank order of potencies was as follows: UDPG ≈ UDP > UTP > UMP. Other nucleoside 5'-*O*-monophates tested (AMP, CMP, GMP, and TMP) had IC_50_ above the concentration range tested (> 20 000 nM) [23].

In this study, we analyzed the potential of various nucleoside 5'-*O*-monophosphorothioates as modulators of the P2Y14 receptor and mast cell degranulation. Stimulation with the tested nucleotides increased cytosolic Ca^2+^ concentration in RBL-2H3 cells suggesting activation of nucleotide receptors. Indeed, Gao et al. [11, 43] demonstrated the existence of receptors for uridine nucleotides (P2Y2, P2Y4, P2Y6, P2Y14) and P2Y receptor subtypes that respond to ADP (P2Y1, P2Y2, P2Y13) on the surface of RBL-2H3 cell line. The P2Y12 receptor was undetectable while the gene expression of the P2Y11R could not be measured, due to the lack of this subtype in rodents./as this subtype is not present in rodents.

Very recently we have shown that pyrimidine nucleoside 5'-*O*-monophosphorothioates are P2Y6 agonists. Although TMPS and UMPS are weaker agonists than uridine 5'-*O*-(2-thiodiphosphate), their P2Y6-dependent increase in HeLa cell migration is comparable with the effect generated by UDPβS [27]. AMPS appeared to be inactive as a P2Y6 agonist, however acted as a partial agonist of P2Y11 receptor [44].

To confirm a direct interaction between the P2Y14 receptor and nucleoside 5'-*O*-monophosphorothioates a stable HEK293T cell line co-expressing the P2Y14R and the chimeric G-protein Gαqi5 was employed. These HEK293T cells responded to AMPS, UMPS, CMPS but not to TMPS with large, concentration-dependent intracellular Ca^2+^ increase. The loss of agonist activity of TMPS molecule can be explained by the fact that 2′ and 3′ hydroxyl groups of the ribose moiety were shown by Trujillo et. al. to be crucial for the P2Y14R activation. Deoxynucleotides are not able to form the necessary H-bonding interactions as in the case of the ribonucleotides [45]. Therefore, to confirm this suggestion, we compared the influence of two adenine-containing phosphorothioates (AMPS and dAMPS) on degranulation of the RBL-2H3 cells and, as it could have been expected, the latter compound was inactive (unpublished data).

In parallel, in a stable P2Y14-expressing HEK293T cell line, thymidine 5'-*O*- monophosphorothioate inhibited UDPG-induced activation of P2Y14R suggesting that it might act as an antagonist. This hypothesis was confirmed with *N*-acetyl-β-D-hexosaminidase release method monitoring mast cell degranulation. TMPS suppressed UDPG-evoked degranulation in antigen-sensitized RBL-2H3 cells. TMPS also inhibited degranulation process stimulated by the selective P2Y14 receptor agonist, namely MRS 2690. At the same time TMPS treatment significantly blunted the increase of intracellular Ca^2+^ concentration evoked by MRS 2690, consistently with an antagonist function of TMPS for the P2Y14R.

Recently, Trujillo et. al. have tried to explain structure-activity relationships of numerous synthetic analogues and their affinity to P2Y14 receptor. In their opinion the P2Y14R binding site contains so called bifurcated binding pocket, where the first subpocket is responsible for the binding of a nucleotide residue and the second one binds glycosyl moiety. According to the authors, the second subpocket is a structurally permissive region able to accept numerous conformations of the pyranose ring [46]. Our results regarding antagonistic properties of TMPS confirm their conclusion on the permissive subpocket, because even without the pyranose ring the binding of a nucleotide and a display of its agonistic or antagonistic activity occurs.

Moreover, Trujillo et al. indicated two amino acid residues (R253 and Q260) as those responsible for the binding of α phosphate group of UGP-glucose [45]. We suggest that one of them (arginine R253) is (can be) involved in hydrogen bonding with sulfur atom of the phosphorothioate group. Some structural studies on phosphorothioate oligonucleotide-proteins complexes indicate an involvement of arginine residue in formation of H•••-S-P hydrogen bonds which are more potent that their H•••-O-P counterparts [46, 47]. Formation of the potent charge-assisted hydrogen bonds which involve negatively charged sulfur atom could explain much higher agonistic or antagonistic activities of nucleoside monophosphorothioates (UMPS, AMPS, TMPS) compared to their unmodified counterparts.

In our opinion, identification of TMPS as the P2Y14 antagonist is an interesting and unexpected finding because even recently it was concluded that nucleotide antagonists of the P2Y14 receptor had not been reported yet [45]. We propose that thymidine 5'-*O*- monophosphorothioate may be a new candidate, especially that it acts as an multifunctional ligand being a partial agonist of the P2Y6R and antagonist of P2Y14. Besides the increased stability of all nucleoside 5'-O-monophosphorothioates as compared to their unmodified counterparts may be responsible for their prolonged action.

## MATERIALS AND METHODS

### Materials and tested compounds

Nucleotides and their phosphorothioate analogues were purchased from the following sources: UDP, UMP and UDPG from Sigma-Aldrich (Saint Louis, MO, USA) and AMPS, CMPS, TMPS, and UMPS from BioLog (Bremen, Germany). The P2Y14 receptor-selective agonist MRS 2690 was provided by Tocris Bioscience (Bristol, UK). Dinitrophenyl conjugated bovine serum albumin (DNP-HSA) and p-nitrophenyl-N-acetyl-β-D-glucopyranoside were obtained from Sigma-Aldrich.

### Cell culture

The rat basophilic leukemia RBL-2H3 cell line was purchased from Leibniz Institute DSMZ - German Collection of Microorganisms and Cell Cultures (Braunschweig, Germany). Cells were cultured in medium consisting of 70% MEM, 20% RPMI 1640, 10% fetal bovine serum containing 100 IU/ml penicillin and 100 μg/ml streptomycin. All reagents for cell culture were obtained from Life Technologies (Carlsbad, CA, USA). Cells were incubated at 37°C in a humidified atmosphere of 95% air and 5% CO_2_.

### Cell viability assay

RBL-2H3 cells were seeded into 96-well plates in the number of 5 × 10^3^ per well in complete medium. After 24 h of incubation, the medium in each well was supplemented with a respective nucleotide added to a final concentration of 100 μM. Cells were incubated in the presence of the investigated compounds for another 24 h. Following incubation, 10 μl of PrestoBlue cell viability reagent (Life Technologies, Van Allen Way, CA, USA), a resazurin-based solution, was added into each well and incubated further for 80 min at 37°C and 5% CO_2_. Cell viability was determined by measuring the fluorescent signal F_530/590_ on a Synergy 2 Microplate Reader (Bio-Rad, CA, USA). The obtained fluorescence magnitudes were used to calculate cell viability expressed as a percent of the viability of the untreated control cells.

### Calcium mobilization assay in HEK293T cells stably expressing the individual P2Y14 receptor

Cell-based assays for activation of the P2Y14 receptor were performed by Multispan Inc. (Hayward, CA, USA). Briefly, HEK293T Gαqi5 cells with stable expression of the P2Y14R were seeded in 384-well black-wall, clear-bottom plates at a density of 20 000 cells per well in 20 μL of growth medium. Ca^2+^ flux assays were conducted after overnight culture according to the manufacturer's protocol using ScreenQuest^TM^Fluo-8 No Wash Calcium Assay kit (AAT Bioquest, Sunnyvale, CA, USA). Dye loading buffer was added to the cells and incubated for 60 minutes at 37°C. Calcium flux was monitored for 90 seconds after compound's application. In the case of antagonist activity, cells were preincubated with the compounds at room temperature for 10 minutes before the application of UDPG serving as the control agonist.

The relative fluorescent units intensity values (Δ RFU) were calculated from maximal fluorescence reading after subtracting the average value of baseline reading.

The percentage of activation by a given compound was calculated from the following equation:

% activation = (ΔRFUCompound −ΔRFUBackground)/(ΔRFUAgonist control - ΔRFUBackground)}•100

### Calcium flux assay in RBL-2H3 cells

For intracellular Ca^2+^measurement RBL-2H3 cells were seeded into 96-well plates (3.5 x10^4^ cells per well) in 100 μl of culture medium and incubated overnight under standard culture conditions. The next day cells were washed two times with 200 μL of Hank's Balanced Salt Solution (Life Technologies) and [Ca^2+^]_i_ level was assessed after stimulation with tested compounds with the Screen Quest^TM^ Fluo-8 No Wash Calcium Assay Kit (AAT Bioquest, Sunnyvale, CA, USA) according to the supplier's protocol. Calcium mobilization was monitored by the change in fluorescence (Ex/Em = 490/520 nm) following stimulation and with respect to the background fluorescence on a Synergy 2 Microplate Reader. In the case of antagonist activity, cells were preincubated with TMPS at room temperature for 10 minutes before the application of MRS 2690 serving as the control P2Y14 agonist.

### Measurement of *N*-acetyl-β-D-hexosaminidase (β-HEX) release from RBL-2H3 cells

RBL-2H3 mast cells suspended in complete growth medium supplemented with DNP-specific IgE antibody (0,5 μg/ml) were spited into 96-well plates and incubated overnight. Then cells were washed two times with 200 μL of Hank's Balanced Salt Solution. Cells were incubated for 20 min with DNP-HSA (10 ng/ml) in the absence or presence of the compounds tested. In the case of antagonist activity, cells were preincubated with the compound at room temperature for 10 minutes before the application of UDPG and MRS 2690 serving as the control agonists.

The *N*-acetyl-β-D-hexosaminidase enzymatic activity of the supernatants and cell lysates (after addition of 0.1% Triton X-100) was determined using *p*-nitrophenyl-*N*-acetyl-β-D-glucopyranoside (8 mM) in 0.08 M citric buffer (pH 4.5) as chromogenic substrate. The enzymatic reaction was stopped by addition of NaOH. The experimental results were gathered by absorbance measurements at 405 nm *vs.* 492 nm using a Synergy 2 Microplate Reader. β-HEX release was calculated using the following formula: Release (%) = (Supernatant − Blank)/(Total − Blank) × 100 and is presented after subtraction of the spontaneous release.

### Statistical analysis

All data are presented as the mean ± S.D. Levels of significance were analyzed by one-way ANOVA with Bonferroni post-test. Statistical significance was determined when P-value was less than 0.05. Differences between groups were rated significant at a probability error *P* < 0.05 (*), *P* < 0.01 (**), and *P* < 0.001 (***).

## SUPPLEMENTARY MATERIAL



## List of abbreviations

AMPS: adenosine 5′-*O*-monophosphorothioate
CMPS: cytidine 5′-*O*-monophosphorothioate
FcεRI: high-affinity immunoglobulin E receptor
DNP-HSA: dinitrophenyl conjugated human serum albumin
β-HEX: *N*-acetyl-β-D-hexosaminidase
MCs: mast cells
MRS 2690: (diphosphoric acid 1-α-D-glucopyranosyl ester 2-[(4′-methylthio)uridin-5′′-yl] ester)
PPTN: 4-((piperidin-4-yl)-phenyl)-7-(4-(trifluoromethyl)-phenyl)-2-naphthoic acid
TMPS: thymidine 5′-*O*-monophosphorothioate
UDPG: UDP-glucose
UMPS: uridine 5′-*O*-monophosphorothioate
[Ca^2+^]_i_: intracellular Ca^2+^ concentration
