# Supplementary material for: Nucleoside 5′-O-monophosphorothioates as modulators of the P2Y14 receptor and mast cell degranulation
Source: Oncotarget. 2016 Oct 9;7(43):69358–70. doi: 10.18632/oncotarget.12541 (PMC5342483; doi:10.18632/oncotarget.12541)
Supplement: Supplementary file 1 [file oncotarget-07-69358-s001.pdf]

Nucleoside 5′-*O*-monophosphorothioates as modulators of the P2Y14 receptor and mast cell degranulation

Supplementary Material

Figure S1

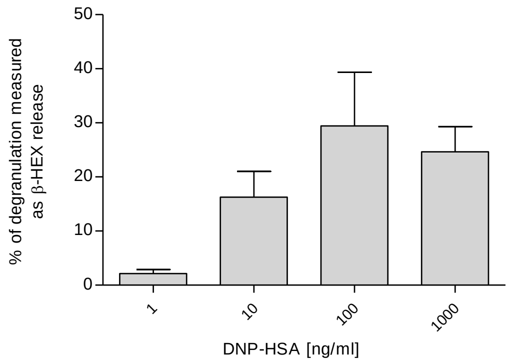

Figure S1: Concentration-dependent influence of DNP-HSA antigen on β-HEX release from RBL-2H3 cells. Mast cells were sensitized for 24 h with DNP-specific IgE antibody (0.5 µg/ml) and incubated for 20 min with antigen. The spontaneous release (9.5%) was subtracted from the final data. Data represent the means ± SD from at least three independent experiments.
